# Pregnancy in the Vagina

**Published:** 1844-01-27

**Authors:** 


### PREGNANCY IN THE VAGINA.

One of the German journals reports a case of extra-uterine gestation in which the fœtus was developed in the vagina. A circumscribed enlargement was apparent between the navel and the pubis, and the bowels and bladder were evacuated with much difficulty. A practitioner appears to have been first called in at about the fourth month (die Geburt schon bis zur *vierten period* vorg. war,) who found the fœtus in a cross position and dead. He immediately proceeded to delivery by the feet, and after much difficulty brought the shoulders through the vulva, and afterwards extracted the head with the forceps. The circumscribed tumour was yet unreduced, and on examination this was found to be due to the uterus itself. That organ was retroverted, its orifice being directed forwards to the abdominal integuments, and closely embracing the cord. The accoucheur contrived, however, to introduce some of his fingers within the os tincæ and remove the placenta, which is said to have been adherent to the neck, and, indeed, to all the rest of the internal surface of the uterus. The woman recovered satisfactorily. We know of only one other recorded case of this very rare kind of extra-uterine gestation; it is detailed in the "Journ. de Med.," &c., of Paris, 1779. The latter case terminated unfavourably to the mother.—*Ibid, from Oest. Wochensch.*

